# Advantages and Inconveniences of a Multi-Agent Large Language Model System to Mitigate Cognitive Biases in Diagnostic Challenges

**DOI:** 10.2196/69742

**Published:** 2025-01-20

**Authors:** Cedric Bousquet, Divà Beltramin

**Affiliations:** 1 Laboratory of Medical Informatics and Knowledge Engineering in e-Health Inserm Sorbonne University Paris France; 2 Public Health and Medical Information Unit Saint-Étienne University Hospital Center Saint-Etienne France; 3 Medical Information Department Civil Hospices of Lyon Lyon France

**Keywords:** large language model, multi-agent system, diagnostic errors, cognition, clinical decision-making, cognitive bias, generative artificial intelligence

We read with great interest a recent article in the *Journal of Medical Internet Research* entitled “Mitigating Cognitive Biases in Clinical Decision-Making Through Multi-Agent Conversations Using Large Language Models: Simulation Study” by Ke et al [1]. Large language models (LLMs) have advanced reasoning skills but most studies have focused on assessing their ability to answer questions [2]. Ke et al [1] evaluated the ability of LLMs to avoid reproducing certain biases that physicians experience when making a diagnosis. The authors simulated decision-making using a multi-agent system in which each agent models the reasoning of a physician using an LLM. The experiment shows a significant increase in performance compared to a human, with an odds ratio of 3.91, which is highly remarkable. This is made possible by a particularly low score for humans, which leaves considerable room for improvement by LLMs.

Previously published studies have often shown disappointing results using LLMs, with performance levels that give cause for concern when used in a clinical context. Some studies have observed performance similar to that of physicians and marginally better in a limited number of cases. We are thus surprised to see that the multi-agent approach has led to such superiority compared to the physicians. This superiority may be explained by using a multi-agent framework, but it could also be limited to the context of addressing cognitive biases.

To investigate this hypothesis, it would be interesting to compare the performance of the multi-agent system with that of a single LLM. In particular, a prompt engineering method known as tree of thought allows several reasoning paths to be explored, enabling the initial choice to be modified according to diverse alternatives [3].

Furthermore, a recent study found no added value in associating an LLM with a physician to improve performance when making a diagnosis [4]. Moreover, the quantity of text and the number of interventions produced by multi-agent systems may be overwhelming, and the physician could ignore the suggestions. This was the case for many studies on computerized decision support systems for drug prescriptions, where too many alerts have led physicians to override relevant alerts [5].

The multi-agent system can lead to significantly higher costs and requires the mobilization of a greater number of resources. This depends on the number of agents involved in the multi-agent system and, above all, on the amount of text generated. The multi-agent system is likely to produce numerous arguments and counterarguments to make a fair diagnosis. It would be interesting to know how many tokens are produced in the application logs compared to a simpler application.

The authors suggest that similar approaches could revolutionize medical practice by making clinical decisions more reliable and more consistent with the levels of evidence available. We subscribe to the idea that LLMs could help physicians, and the authors’ approach seems promising. However, there is still a lack of evidence on how these multi-agent systems would perform if they were generalized to other diagnostic challenges.

## Abbreviations

LLM: large language model
